# Prognostic and Clinicopathological Significance of MUC Family Members in Colorectal Cancer: A Systematic Review and Meta-Analysis

**DOI:** 10.1155/2019/2391670

**Published:** 2019-12-20

**Authors:** Chao Li, Didi Zuo, Tao Liu, Libin Yin, Chenyao Li, Lei Wang

**Affiliations:** ^1^Department of Colorectal and Anal Surgery, The First Hospital of Jilin University, Changchun, China; ^2^Department of Endocrinology and Metabolism, The First Hospital of Jilin University, Changchun, China

## Abstract

**Objective:**

To assess the association between MUC expression levels in colorectal cancer (CRC) tissues and prognosis and investigate the associations between MUC expression levels and CRC clinicopathological characteristics.

**Methods:**

The PubMed, Embase, Cochrane Library, and Web of Science databases were searched from inception through September 13, 2019, to identify studies investigating the association between MUC expression levels in CRC tissues and prognosis. Pooled hazard ratios (HRs) or odds ratio (ORs) with 95% confidence intervals (CIs) were used to evaluate associations between MUC expression levels and prognosis or clinicopathological characteristics, respectively. The heterogeneity between studies was assessed by the *I*^2^ values, whereas the likelihood of publication bias was assessed by Egger's linear regression and Begg's rank correlation test.

**Results:**

Among 33 included studies (*n* = 6032 patients), there were no associations between combined MUC phenotype expression levels and overall survival (OS) or disease-free survival (DFS)/relapse-free survival (RFS) in patients with CRC. In subgroup analyses, the upregulated MUC1 expression (HR = 1.50; 95% CI, 1.29–1.74; *P* < 0.00001) was associated with poor OS. However, the upregulated MUC2 expression (HR = 0.64; 95% CI, 0.52–0.79; *P* < 0.00001) was associated with better OS. Furthermore, a high level of MUC1 expression (HR = 1.99; 95% CI, 0.99–3.99; *P* = 0.05) was associated with shorter DFS/RFS. However, patients with a low level of MUC2 tumors showed better DFS/RFS than patients with a high level of MUC2 tumors (HR = 0.71; 95% CI, 0.49–1.04; *P* = 0.08; *P* = 0.0.009, *I*^2^ = 67%) and MUC5AC expression (HR = 0.56; 95% CI, 0.38–0.82; *P* = 0.003) was associated with longer DFS/RFS. In addition, a high level of MUC1 expression was associated with CRC in the rectum, deeper invasion, lymph node metastasis, distant metastasis, advanced tumor stage, and lymphatic invasion. A high level of MUC2 expression had a protective effect. High secretion of MUC5AC is associated with colon cancer compared with rectal cancer.

**Conclusion:**

The protein expression of MUC1 might be a poor biomarker in colorectal cancer and might play a role in tumor transformation and metastasis. However, the protein expression of MUC2 expression might have a protective effect. Furthermore, randomized controlled trials (RCTs) of large patients are needed to confirm the results.

## 1. Introduction

Colorectal cancer (CRC) is among the most frequently diagnosed cancers in the United States (US) [1]. In 2018, an estimated 140,250 Americans will be diagnosed with CRC and 50,630 individuals will die from the disease [2]. Although morbidity and mortality in CRC are reduced by high-quality healthcare and healthy lifestyles, the 5-year overall survival (OS) rates after initial diagnosis remain at 67% for patients with rectal cancer and 64% for patients with colon cancer [1]. Furthermore, CRC survivors have a high risk of cancer recurrence [3, 4] and secondary tumors, particularly in the digestive system [5].

The classic tumor, node, and metastasis (TNM) staging system is regarded as the standard prognostic parameter and forms the basis for treatment decisions in CRC [6]. However, since the TNM system fails to reflect the intrinsic biological heterogeneity of CRC, especially in patients with atypical early or occult metastases, only 40% of CRCs are diagnosed at an early stage and approximately 50% of recently diagnosed cases will progress to metastatic cancer [7]. In addition, the prognostic value of TNM in patients with CRC is suboptimal [8]. Currently, there is an unmet need for biomarkers that accurately predict CRC progression, metastasis, and treatment outcomes [9].

In recent years, increasing attention has been given to the role of mucins (MUC) in the pathogenesis of cancer. MUC are a family of high molecular weight glycosylated proteins [10], which have a highly polymorphic tandem repeat in the central region [11]. At present, approximately 20 MUC have been identified. These can be divided into two major subfamilies, secreting gel-type mucins and transmembrane mucins, according to their structure and function [12]. MUC are usually expressed on the apical surfaces of normal glandular epithelial cells and luminal epithelial cells and have key functions in immunity, cell adhesion, and intracellular signaling [13]. Studies on the subcellular distribution of MUC and biochemical characteristics of malignant transformation and progression implicate MUC in tumorigenesis and metastasis [14–18], suggesting that abnormal MUC expression may be a predictive biomarker of CRC.

Evidence suggests that MUC expression is involved in the invasion and metastasis of various malignancies, including gallbladder cancer [19], breast cancer [20], ovarian cancer [21], gastric carcinoma [22, 23], pancreatic carcinoma [24–26], ampullary cancer [27, 28], lung cancer [16, 29], prostate cancer [30], renal cell carcinoma [31], and appendiceal carcinoma [32]. However, the prognostic value of MUC expression in CRC remains controversial [33–37]. To clarify the inconsistent findings from previously published studies investigating the role of MUC in CRC, this meta-analysis was conducted to assess the association between MUC expression levels and prognosis in CRC and investigate the associations between MUC expression levels and several CRC clinicopathological characteristics.

## 2. Materials and Methods

This systematic review and meta-analysis is reported according to the Preferred Reporting Items for Systematic Reviews and Meta-Analyses (PRISMA) guideline [38]. Basing on previously published studies, our study does not include any research with humans or animals, so ethical recognition and patient consent are not required.

### 2.1. Search Strategy

Two review authors independently searched the PubMed, Embase, Cochrane Library, and Web of Science databases from inception through September 13, 2019. Keywords included (“mucins” OR “mucin” OR “MUC”) AND (“colorectal cancer” OR “colorectal neoplasm” OR “colorectal tumor” OR “colonic cancer” OR “colon cancer” OR “rectal cancer” OR “CRC”) AND (“prognostic “OR “prognosis” OR “outcome” OR “survival”). A manual search of the reference lists of relevant articles was performed. Searches were limited to articles published in English or Chinese language.

### 2.2. Inclusion and Exclusion Criteria

The inclusion criteria were (1) study design: cohort study; (2) population: patients with CRC; (3) parameter: MUC expression levels in CRC tissues; and (4) outcome: association between MUC expression levels in CRC tissues and prognosis.

The exclusion criteria were as follows: (1) duplicate publications; (2) in vitro or animal studies; (3) reviews, conference reports, meta-analyses, books, case reports, or letters; or (4) studies that reported insufficient data. When articles reported data from the same study, the most recent article was included.

### 2.3. Data Extraction

Two review authors independently extracted data from the eligible studies, including the surname of the first author, year, country, sample size, patients' mean age, MUC phenotype, antibody for MUC, cut-off value for MUC, frequency of high MUC expression, detection method, TNM stage, histologic type, mean tumor dimensions, median follow-up, and outcomes. Disagreements about data extraction were resolved by discussion with a third reviewer until consensus was reached.

### 2.4. Quality Assessment

Two review authors independently conducted an assessment of the methodological quality of included studies using the Newcastle Ottawa Scale (NOS) [39]. The NOS assessed the quality of the enrolled groups, the comparability and outcomes of the study populations, and study quality on a scale from 0 to 9 points, with ≥7 considered high-quality research.

Publication bias was evaluated using Egger's linear regression and Begg's rank correlation test [40].

### 2.5. Statistical Analysis

Statistical analyses were performed using Review Manager, version 5.3 (Cochrane Collaboration, Copenhagen, Denmark) and STATA, version 12.0 (Stata Corporation, College Station, TX, USA). Survival analysis was performed according to Moher et al. [38]. Hazard ratios (HRs) were directly extracted from included studies, or digitized and extracted using Engauge Digitizer version 4.1 (http://markummitchell.github.io/engauge-digitizer/) software when prognostic information was plotted as a Kaplan-Meier curve [41]. Pooled HRs with corresponding 95% confidence intervals (CIs) were used to assess the association between MUC expression levels (low vs. high) in CRC tissues and OS or disease-free survival (DFS)/relapse-free survival (RFS). Odds ratios (ORs) with 95% CIs were used to assess the impact of MUC expression levels on clinicopathological characteristics.

Studies with significant heterogeneity were identified with the chi-squared test (*P* ≤ 0.10) and the inconsistency index (*I*^2^ ≥ 50%) [42]. When significant heterogeneity was found, a random effects model was adopted. Otherwise, a fixed effects model is used. Subgroup analyses stratified by MUC phenotype and metaregression analysis were performed to explore sources of heterogeneity. The likelihood of publication bias was assessed by Egger's linear regression and Begg's rank correlation test. Sensitivity analysis evaluated the robustness of the data by omitting one study at a time. *P* < 0.05 was considered statistically significant.

## 3. Results

### 3.1. Search Results

**Figure 1:**
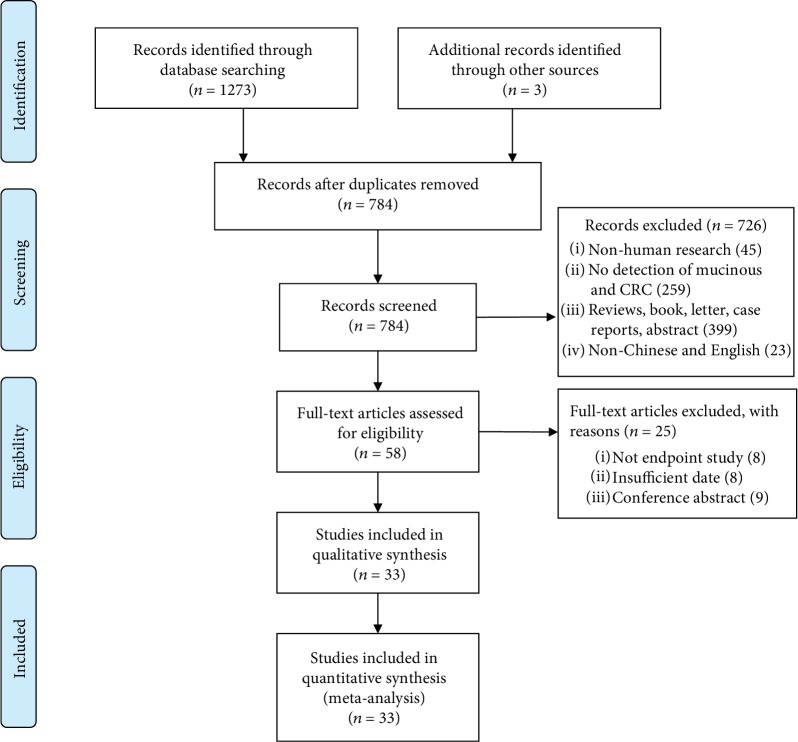
Flow diagram of included studies.

A total of 1273 articles were identified from the electronic search of the databases, and 3 additional studies were obtained from the manual search of the reference lists of relevant articles. After excluding 492 duplicates, titles and abstracts were screened, and 726 studies that did not meet the inclusion criteria were excluded. The full text of 58 studies was retrieved for further review, and 8 articles that did not report an endpoint, 8 articles with insufficient data, and 9 conference abstracts were excluded. Finally, 33 observational studies [33–37, 43–70] were found eligible for inclusion in our review (Figure 1).

### 3.2. Characteristics of the Included Studies

The characteristics of the included studies are shown in Table 1. The 33 eligible studies were published between 1987 and 2019. The studies included a total of 6032 cases. The mean age of patients ranged from 54.3 to 72.0 years, and the median follow-up ranged from 18.0 to 116.0 months. All included studies evaluated the correlation between MUC expression levels in CRC tissues and prognosis. 31 studies evaluated MUC expression using immunohistochemistry (IHC), and 2 studies used reverse transcriptase polymerase chain reaction (qRT-PCR). Nine MUC phenotypes, determined by the expression of MUC1, MUC2, MUC3, MUC4, MUC5AC, MUC12, MUC16, MUC20, and sialomucin, were associated with prognosis in CRC. Various anti-MUC monoclonal antibodies were utilized to identify the MUC phenotypes, and each study applied a different cut-off point (low/high level) to assess MUC expression.

### 3.3. Methodological Quality

According to the NOS, all included studies were of high methodological quality (score ≥ 7) ([Supplementary-material supplementary-material-1]).

### 3.4. MUC Expression and Overall Survival in CRC

**Figure 2:**
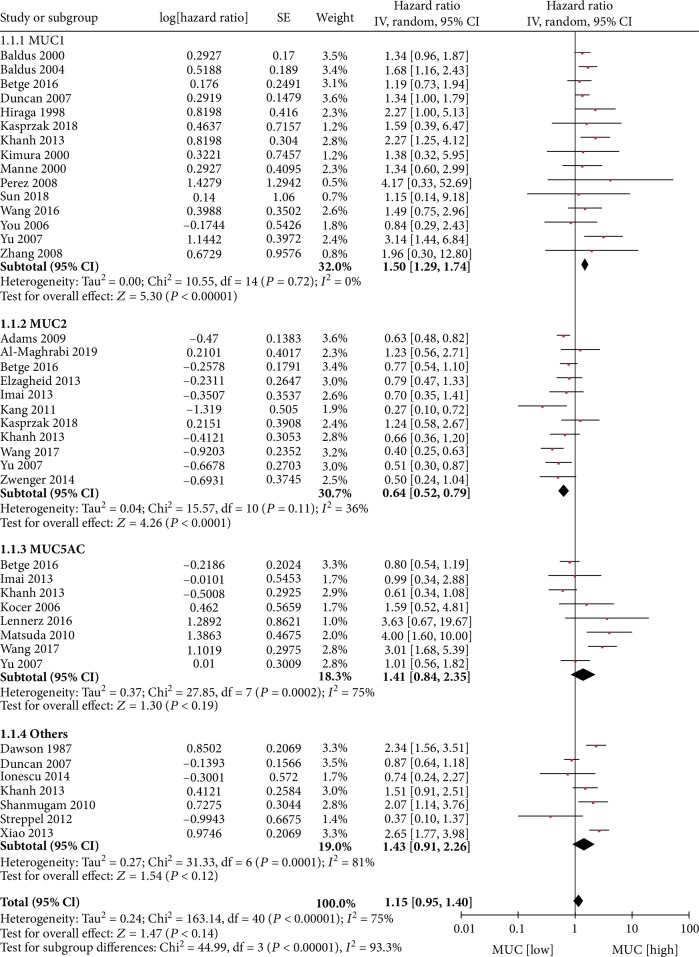
MUC expression and OS.

The association between MUC expression levels in CRC tissues and OS was investigated in 41 datasets from 30 articles; each dataset represented various MUC phenotypes. The meta-analysis demonstrated no association between combined MUC phenotype expression levels and OS (HR = 1.15; 95% CI, 0.95–1.40; *P* = 0.14). There was evidence of significant heterogeneity between studies (*P* < 0.00001, *I*^2^ = 75%). The source of the heterogeneity was investigated in a subgroup analysis stratified by specific MUC phenotype. The subgroup analysis demonstrated that a high level vs. a low level of MUC1 expression (HR = 1.50; 95% CI, 1.29–1.74; *P* < 0.00001; *P* = 0.72, *I*^2^ = 0%) or a low level vs. a high level of MUC2 expression (HR = 1.56; 95% CI, 1.27–1.92; *P* < 0.00001; *P* = 0.11, *I*^2^ = 36%) was associated with poor OS in patients with CRC. However, associations between the levels of MUC5AC (HR = 1.41; 95% CI, 0.84–2.35; *P* = 0.19; *P* = 0.0002, *I*^2^ = 75%), other MUC phenotypes (HR = 1.43; 95% CI, 0.91–2.26; *P* = 0.12; *P* < 0.00001, *I*^2^ = 81%), and OS were not significant (Figure 2).

### 3.5. MUC Expression and Disease-Free Survival/Recurrence-Free Survival in CRC

**Figure 3:**
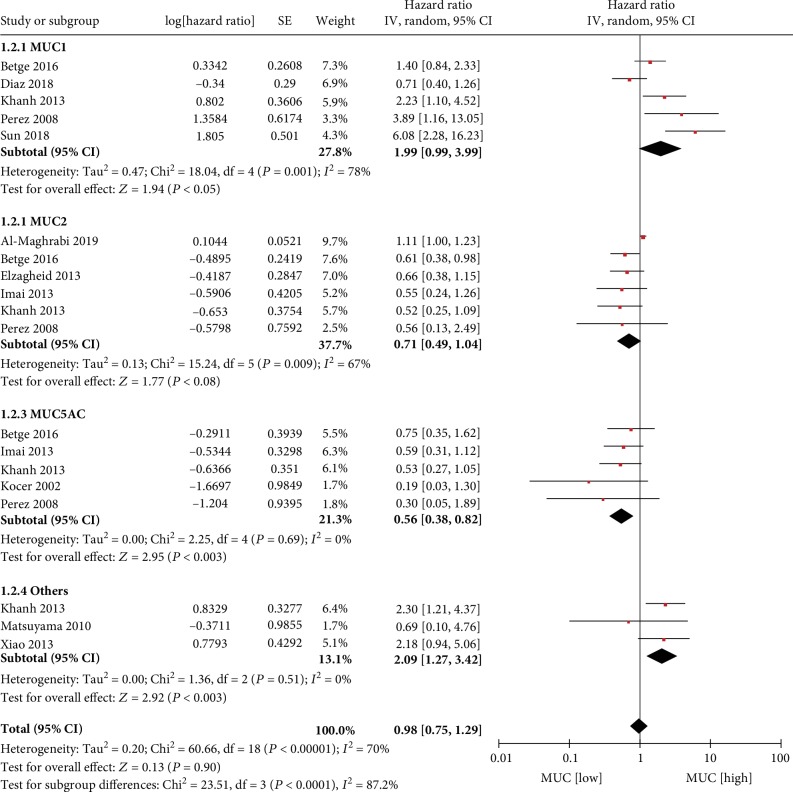
MUC expression and DFSRFS.

The association between MUC expression level in CRC tissues and DFS/RFS was investigated in 19 datasets from 11 articles. The meta-analysis demonstrated no association between combined MUC phenotype expression levels and DFS/RFS (HR = 0.98; 95% CI, 0.75–1.29; *P* = 0.90). There was evidence of significant heterogeneity between studies (*P* < 0.00001, *I*^2^ = 70%). The source of the heterogeneity was investigated in a subgroup analysis stratified by specific MUC phenotype. The subgroup analysis demonstrated that a high level vs. a low level of MUC1 expression (HR = 1.99; 95% CI, 0.99–3.99; *P* = 0.05; *P* = 0.0001, *I*^2^ = 78%) or other MUC expression (HR = 2.09; 95% CI, 1.27–3.42; *P* = 0.003; *P* = 0.51, *I*^2^ = 0%) was associated with shorter DFS/RFS in patients with CRC. However, a high level vs. a low level of MUC5AC expression (HR = 0.56; 95% CI, 0.38–0.82; *P* = 0.003; *P* = 0.69, *I*^2^ = 0%) was associated with longer DFS/RFS and patients with a low level of MUC2 tumors showed better DFS/RFS than patients with a high level of MUC2 tumors (HR = 0.71; 95% CI, 0.49–1.04; *P* = 0.08; *P* = 0.0.009, *I*^2^ = 67%).(Figure 3).

### 3.6. MUC Expression and CRC Clinicopathological Characteristics

The meta-analysis demonstrated no association between combined MUC phenotype expression levels and CRC clinicopathological characteristics. In all analyses, there was evidence of significant heterogeneity between studies. The source of the heterogeneity was investigated in subgroup analyses stratified by specific MUC phenotype (Table 2).

A high level of MUC1 expression (III/IV vs. I/II: OR = 2.17, 95% CI = 1.31–3.59, *P* = 0.002) was associated with advanced tumor stage in patients with CRC than MUC2 expression (III/IV vs. I/II: OR = 0.52, 95% CI = 0.36–0.76, *P* = 0.0008), but the association between MUC5AC expression and tumor stage was not significant.

A high level of MUC1 expression (T3/T4 vs. T1/T2: OR = 1.79, 95% CI = 1.41–2.26, *P* < 0.00001) was associated with deeper invasion in patients with CRC, but the association between MUC5AC and MUC2 expression and depth of invasion was not significant.

A high level of MUC1 expression (positive vs. negative: OR = 2.45, 95% CI = 1.38–4.35, *P* = 0.002) was associated with lymph node metastasis in patients with CRC than MUC2 expression (positive vs. negative: OR = 0.59, 95% CI = 0.47–0.73, *P* < 0.00001), but the association between MUC5AC expression and lymph node metastasis was not significant.

A high level of MUC1 expression (positive vs. negative: OR = 0.79, 95% CI = 0.63–0.98, *P* = 0.03) was associated with rectum cancer. However, the elevated MUC2 expression (positive vs. negative: OR = 1.64, 95% CI = 1.01–2.67, *P* = 0.04) and MUC5AC expression (positive vs. negative: OR = 1.97, 95% CI = 1.48–2.62, *P* < 0.00001) were associated with colon cancer.

A high level of MUC1 expression was associated with distant metastasis (positive vs. negative: OR = 2.47, 95% CI = 1.47–4.13, *P* = 0.0006) and lymphatic invasion (positive vs. negative: OR = 3.39, 95% CI = 1.69–9.14, *P* = 0.001) in patients with CRC. A high level of MUC2 expression was associated mucinous cancer (high vs. low: OR = 14.46, 95% CI = 1.71–121.97, *P* = 0.01) and low histological grade (3 vs. 1 and 2: OR = 0.75, 95% CI = 0.56–0.99, *P* = 0.04).

There were no associations between the expression levels of any MUC phenotypes and other clinicopathological characteristics, including gender or tumor size.

### 3.7. Sensitivity Analysis and Publication Bias

**Figure 4:**
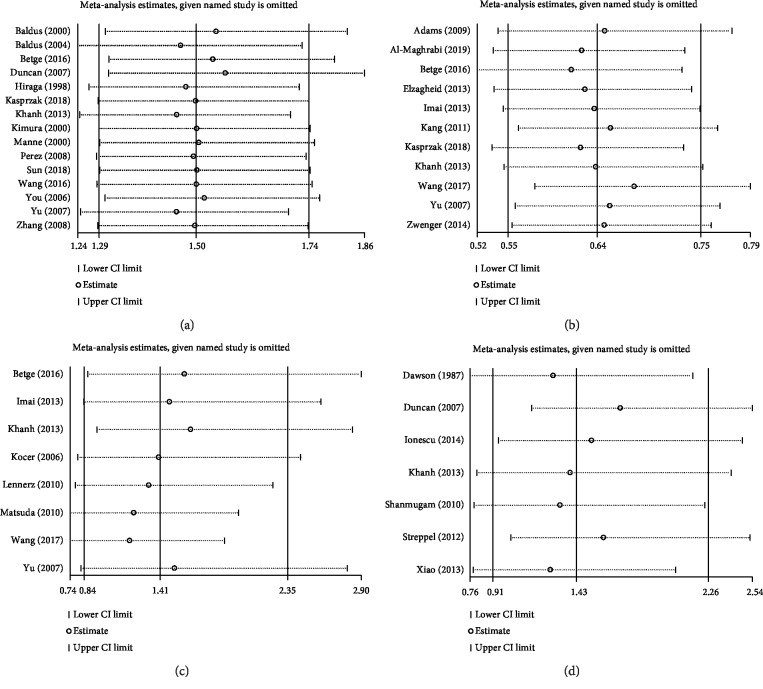
Sensitivity analysis for MUC expression ((a): MUC1, (b): MUC2, (c): MUC5AC, (d): Others MUC) and OS.

**Figure 5:**
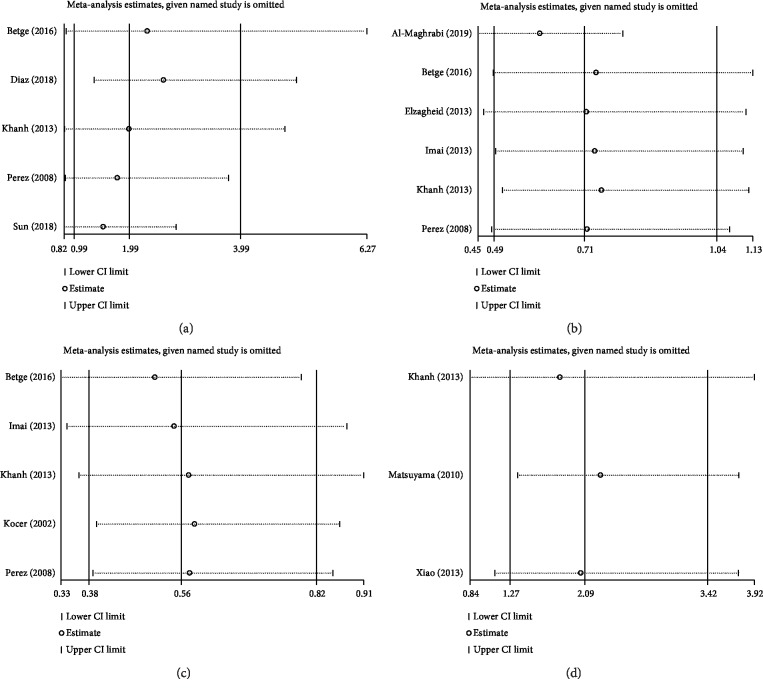
Sensitivity analysis for MUC expression ((a): MUC1, (b): MUC2, (c): MUC5AC, (d): Others MUC) and DFS/RFS.

**Figure 6:**
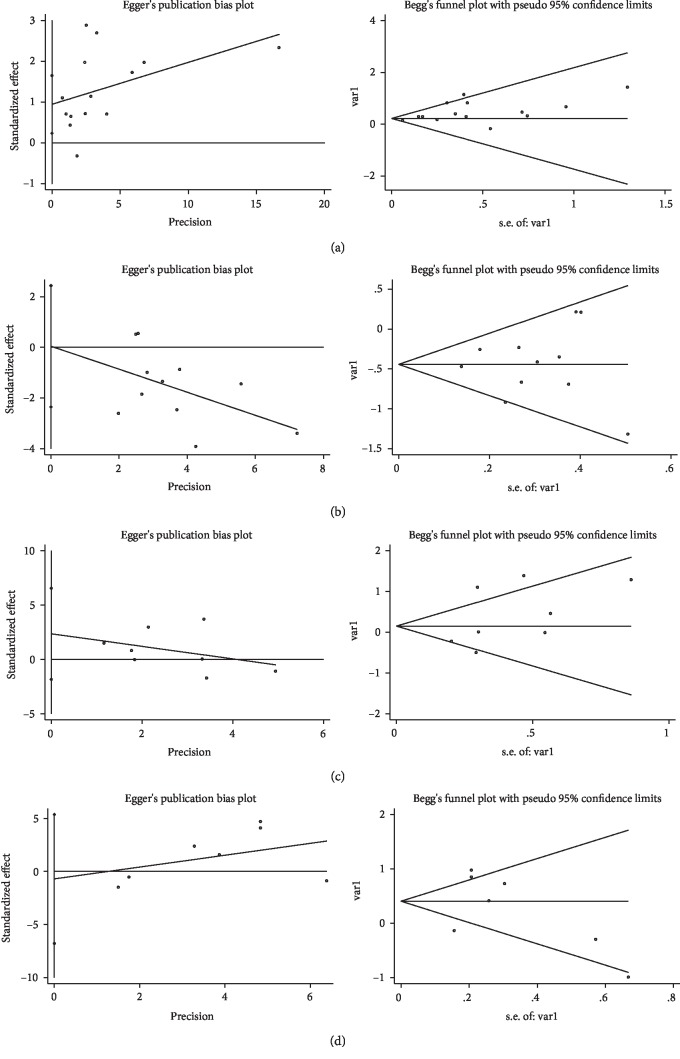
Publication bias for MUC expression ((a): MUC1, (b): M UC2, (c): MUC5AC, (d): Others MUC) and OS.

**Figure 7:**
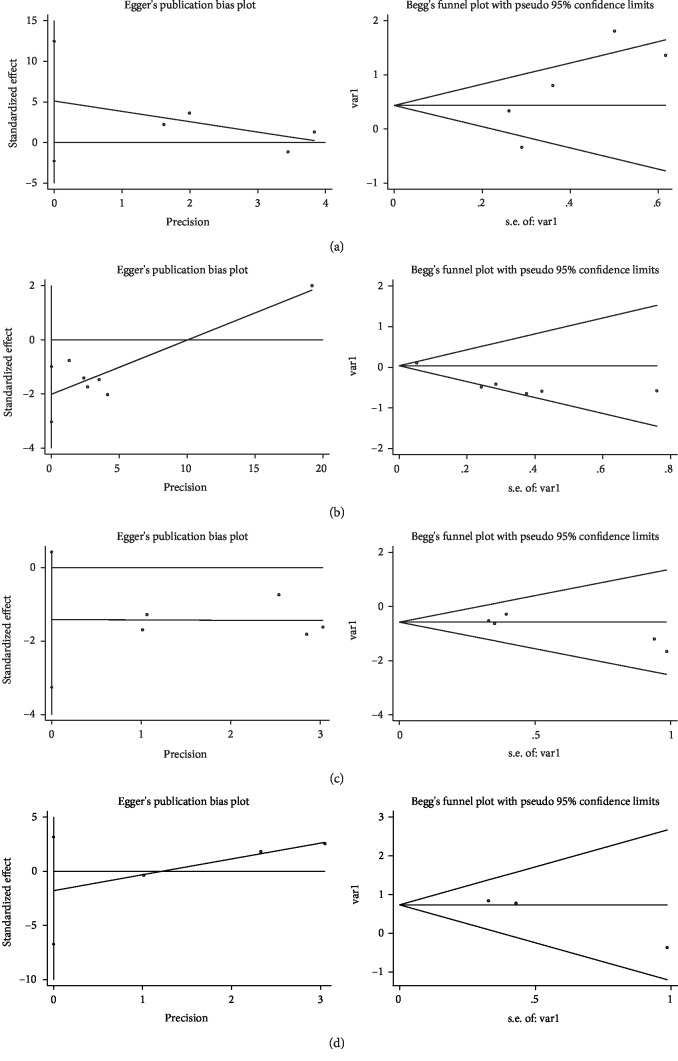
Publication bias for MUC expression ((a): MUC1, (b): MUC2, (c): MUC5AC, (d): Others MUC) and DFS/RFS.

Sensitivity analysis omitting one study at a time demonstrated the associations of MUC family members' expression with OS (Figure 4) and DFS/RFS (Figure 5) in CRC were robust. Begg's rank correlation test and Egger's linear regression showed no publication bias among studies investigating OS (Figure 6) and DFS/RFS (Figure 7).

### 3.8. Metaregression

Metaregression was performed to explore the factors influencing the association of MUC expression with OS and DFS/RFS in CRC. None of the covariates (cut-off value, antibody, TNM stage, country, and years) analyzed were identified as potential sources of heterogeneity (Table 3).

## 4. Discussion

In this meta-analysis, we assessed the association between MUC expression levels in CRC tissues and prognosis and investigate the associations between MUC expression levels and several CRC clinicopathological characteristics. Interestingly, findings demonstrated no association between combined MUC phenotype expression levels in CRC tissues and prognosis. However, in subgroup analyses stratified by MUC phenotype, a high level of MUC1 expression was associated with poor OS and DFS/RFS, a high level of MUC2 expression was associated with improved OS and DFS/RFS, and a high level of MUC5AC was associated with improved DFS/RFS. Generally, heterogeneity between studies was significantly reduced in the subgroup analyses stratified by MUC phenotype. Meanwhile, meta-regression analysis revealed that antibody for MUC, cut-off value for MUC, TNM stage, and histologic type were not significant sources of heterogeneity.

However, importantly, several studies have shown a correlation between MUC expression and patient with various cancers. For example, a meta-analysis reported that MUC expression was significantly higher in patients with esophageal adenocarcinoma than in normal squamous esophageal mucosa [71]. The study by Lu et al. [72] also indicated that increased MUC expression was associated with worse OS and more detrimental clinicopathological outcomes in head and neck cancer patients. Overall, it is reasonable that the expression of MUC was associated with variable clinical outcomes in different tumors. These differences may be due to different mechanisms, pathways, and treatment options. An earlier meta-analysis have shown that abnormal expression of MUC in CRC tissues compared with healthy mucosa plays an important role in the pathogenesis and progression of CRC [73]. Several meta-analyses have explored the association between MUC expression and CRC clinicopathological characteristics [74–76]. Furthermore, compared with two earlier meta-analyses for various types of cancer by Xu et al. [77] and Huang et al. [78], the present analysis not only added additional 26 and 27 studies in colorectal cancer subtype but also examined the correlation between MUC expression and the clinicopathological factors of colorectal cancer.

The current study explored the association between MUC expression levels in CRC tissues and CRC clinicopathological characteristics. A high level of MUC1 expression was associated with CRC in the rectum, deeper invasion, lymph node metastasis, distant metastasis, advanced tumor stage, and lymphatic invasion. Elevated MUC2 expression was associated with CRC in the colon, shallower lesions, negative lymph node metastasis, early stage of tumor, mucinous carcinoma, and larger tumor size. MUC5AC was more easily expressed in colon cancer. These findings implicate MUC1 in mechanisms that promote tumor invasion, lymph node metastasis, high stage, lymphatic invasion, and poor survival in CRC, while MUC2 may have a protective role. A number of studies have demonstrated a unique role for MUC in proliferation, survival, metastasis, epithelial-mesenchymal transition, and antiapoptosis in tumors [13, 17, 79–82]. As a ligand of cell adhesion molecules, MUC 1 induces circulating tumor cells (CTCs) to adhere to endothelial cells or transport to distant sites, establishing secondary tumors [81]. MUC2 is major structural component of the inner mucus layer in the colon, which is impervious to bacteria and protects the colon epithelium. Decreased MUC2 expression allows bacteria to contact the epithelial surface, triggering inflammatory bowel disease, which can lead to colon cancer [83]. Studies characterizing the function of MUC5AC are scarce. Hoshi et al. [84] showed that MUC5AC protects pancreatic cancer cells from TRAIL-induced apoptosis, while other reports suggest that MUC5AC has no effect on cell growth, cell survival, proliferation, or morphology in vitro [85].

Findings from the current meta-analysis indicate MUC1 may be a biomarker of poor prognosis in CRC and suggest that combined detection of MUC1 and MUC2 should be used to accurately predict CRC progression, metastasis, and treatment outcomes. Understanding the association between MUC expression levels and metastasis in CRC may help clarify the risk of metastasis at the time of diagnosis in patients with CRC, especially in those patients without symptoms or signs of metastasis. Clinically, MUC detection is simple and easy to implement.

This study was associated with several limitations. First, HRs from some of the included studies were calculated from Kaplan-Meier curves, which may have influenced the robustness of our findings. Second, the lack of a standardized detection methods and antibodies to detect MUC status may have affected the accuracy of our results. Third, despite the use of subgroup analysis and meta-regression to identify potential sources of heterogeneity between studies, they may have been additional sources of heterogeneity that impacted our findings. Finally, the sample size was small, and results should be considered preliminary.

In conclusion, findings from the current study suggest that MUC1 and MUC2 expression levels in CRC tissues are associated with OS, DFS/RFS, tumor site, depth of invasion, lymph node metastasis, distant metastasis, tumor stage, histologic type, and lymphatic invasion. These results indicate that MUC status can be used to differentiate between normal cells and CRC cells and predict a patient's clinicopathological characteristics and prognosis. The clinical relevance of MUC regulation in CRC tissues remains to be elucidated in large well-designed cohort studies.

## Figures and Tables

**Table 1:** Characteristics of the included studies.

First author	Year	Country	Patient number	Detection method	Mean age (years)	Media follow-up (mounts)	Outcome	Mucins phenotype	Antibody	Cut-off value (high level)	High MUC expression
Adams	2009	Switzerland	938	IHC	70.5	128.0	OS	MUC2	NR	PP > 5%	NR
Al-Maghrabi	2019	Saudi Arabia	128	IHC	NR	NR	OS/DFS	MUC2	MRQ-18	PP ≥ 25%	36.7%
Baldus	2000	Germany	264	IHC	64.8	NR	OS	MUC1	NCL-MUC1	PP > 5%	58.0%
Baldus	2004	Germany	205	IHC	65.0	NR	OS	MUC1	HMFG-2	PP > 35%	49.8%
Betge	2016	Germany	381	IHC	68.5	NR	OS/DFS	MUC1	Ma695	PP > 0%	64.0%
								MUC2	Ccp-58	PP > 0%	77.0%
								MUC5AC	45M1	PP > 0%	48.9%
								MUC6	MCN6.01	PP > 0%	28.7%
Dawson	1987	UK	358	IHC	65.7	18.0	OS	Sialomucin	High iron diamine-alcian blue	Blue staining	29.6%
Diaz	2018	Spain	96	IHC	65.9	NR	DFS	MUC1	Clone E29	PP ≥ 50%	46.0%
Duncan	2007	UK	403	IHC	72.0	116.0	OS	MUC1	Ma695	PP ≥ 30%	31.5%
								MUC3	1143/B7	PP ≥ 30%	73.9%
Elzagheid	2013	Libya	141	IHC	NR	77.0	OS/DFS	MUC2	MRQ-18	PP > 0%	50.0%
Hiraga	1998	Japan	100	IHC	62.7	80.0	OS	MUC1	KL-6	PP > 30%	71.0%
Imai	2013	Japan	250	IHC	66.9	NR	OS/RFS	MUC2	Ccp-58	PP ≥ 25%	49.4%
								MUC5AC	CLH2	PP ≥ 1%	46.8%
Ionescu	2014	Romania	39	qRT-PCR	66.0	NR	OS	MUC12	NR	NR	NR
Kang	2011	Korea	229	IHC	NR	108	OS	MUC2	NR	Score ≥ 6	24.2%
Kasprzak	2018	Poland	34	IHC	NR	NR	OS	MUC1	Ma552	PP ≥ 2.57%	100%
								MUC2	Ccp-58	PP ≥ 4.97%	100%
Khanh	2013	Japan	206	IHC	NR	NR	OS/RFS	MUC1	Ma695	PP ≥ 25%	62.6%
								MUC2	Ccp-58	PP ≥ 50%	32.5%
								MUC4	1G8	PP ≥ 50%	33.0%
								MUC5AC	CLH2	PP ≥ 5%	33.5%
Kimura	2000	Japan	110	IHC	63.1	68.5	OS	MUC1	KL-6	PP ≥ 30%	69.1%
Kocer	2002	Turkey	41	IHC	56.3	NR	DFS	MUC5AC	45M1	ISS > 0.1	34.1%
Kocer	2006	USA	30	IHC	59.0	39.0	OS	MUC5AC	45M1	PP > 10%	60.0%
Lennerz	2016	USA	33	IHC	58.0	51.2	OS	MUC2	Ccp58	PP ≥ 10%	84.0%
								MUC5AC	CLH2	PP ≥ 10%	45.0%
								MUC6	CLH5	PP ≥ 10%	0.0%
Manne	2000	USA	166	IHC	65.3	NR	OS	MUC1	DF3	SI ≥ 0.5	39.8%
								MUC2	Ccp58	SI ≥ 0.5	80.7%
Matsuda	2010	*Japan*	569	IHC	68.0	NR	OS	MUC2	Anti-MUC2	PP ≥ 10%	65.0%
								MUC5AC	Anti-MUC5	PP ≥ 10%	15.1%
								MUC6	Anti-MUC6	PP ≥ 10%	1.9%
Matsuyama	2010	Japan	100	qRT-PCR	65.1	27.0	DFS	MUC12	Rabbit polyclonal antibody	NR	NR
Perez	2008	Brazil	35	IHC	62.2	NR	OS/DFS	MUC1	Ma695	PP > 10%	20.0%
								MUC2	Ccp-58	PP > 10%	65.7%
								MUC5AC	CLH2	PP > 10%	22.9%
Shanmugam	2010	USA	132	IHC	65.0	NR	OS	MUC4	Clone 8G7	ISS > 2	24.2%
Sun	2018	China	118	IHC	54.3	57.0	OS/DFS	MUC1	MXB Biotechnologies	PP ≥ 10%	14.4%
Streppel	2012	USA	39	IHC	63.6	NR	OS	MUC16	Monoclonal antibody	PP > 0%	64.1%
Wang	2016	China	81	IHC	63.5	NR	OS	MUC1	ZM-0391	ISS > 1	53.1%
Wang	2017	China	139	IHC	NR	NR	OS	MUC2	NCL-MUC2	PP > 20%	48.2%
								MUC5AC	NCL-MUC5	PP > 20%	28.1%
Xiao	2013	China	150	IHC	55.0	NR	OS/DFS	MUC20	Mouse antihuman polyclonal antibody	ISS > 2	60.7%
You	2006	China	203	IHC	NR	111.9	OS	MUC1	Ma695	IRS ≥ 2	40.7%
Yu	2007	China	150	IHC	57.5	NR	OS	MUC1	Ma695	ISS ≥ 2	45.3%
								MUC2	Ccp-58	ISS ≥ 2	52.6%
								MUC5AC	45M1	ISS ≥ 2	44.0%
Zhang	2008	Japan	77	IHC	64.9	NR	OS	MUC1	KL-6	SI (positive)	55.8%
Zwenger	2014	Argentina	90	IHC	NR	NR	OS	MUC1	HMFG1	Score > 0	94.0%
								MUC2	H300	Score > 0	52.4%

NR: not reported; RT-PCR: reverse transcriptase polymerase chain reaction; IHC: immunohistochemistry; SI: staining intensity; PP: positive cell percentage; immunostaining score (ISS): PP^∗^SI (while groups I and II (absent and low) were considered negative expression).

**Table 2:** Meta-analysis of the correlation between MUC expression and clinicopathological factors of colorectal cancer.

Clinicopathological parameter	Mucins phenotype	No. of studies	OR (95% CI)	Analysis model	Test for overall effect		Heterogeneity	
					*Z* test	*P* value	*I* ^2^ (%)	*P* value
TNM stage (III/IV vs. I/II)	MUC1	11	2.17 (1.31-3.59)	Random	3.03	0.002	83	<0.00001
	MUC2	7	0.52 (0.36-0.76)	Random	3.35	0.0008	52	0.05
	MUC5AC	8	1.00 (0.67-1.49)	Random	0.01	0.99	55	0.03

Depth of invasion (T3/T4 vs. T1/T2)	MUC1	11	1.79 (1.41-2.26)	Fixed	4.86	<0.00001	40	0.08
	MUC2	6	0.65 (0.37-1.13)	Random	1.53	0.13	63	0.02
	MUC5AC	4	0.64 (0.35-1.18)	Random	1.42	0.15	61	0.05

Lymph node metastasis (+ vs. -)	MUC1	10	2.45 (1.38-4.35)	Random	3.07	0.002	81	<0.00001
	MUC2	8	0.59 (0.47-0.73)	Fixed	4.64	<0.00001	48	0.06
	MUC5AC	7	1.07 (0.67-1.72)	Random	0.29	0.77	67	0.006

Tumor site (colon vs. rectum)	MUC1	7	0.79 (0.63-0.98)	Fixed	2.12	0.03	0	0.63
	MUC2	5	1.64 (1.01-2.67)	Random	2.02	0.04	55	0.06
	MUC5AC	6	1.97 (1.48-2.62)	Fixed	4.63	<0.00001	49	0.08

Distant metastasis (+ vs. -)	MUC1	3	2.47 (1.47-4.13)	Fixed	3.43	0.0006	49	0.14
	MUC2	3	0.83 (0.48-1.41)	Fixed	0.70	0.49	0	0.61
	MUC5AC	2	0.86 (0.15-4.87)	Random	0.17	0.87	73	0.06

Lymphatic invasion (+ vs. -)	MUC1	5	3.39 (1.69-9.14)	Random	3.19	0.001	72	0.007
	MUC2	3	0.53 (0.27-1.03)	Random	1.88	0.06	60	0.08
	MUC5AC	4	0.76 (0.55-1.05)	Fixed	1.64	0.10	20	0.29

Mucinous component (high vs. low)	MUC1	7	0.71 (0.42-1.19)	Random	1.31	0.19	59	0.02
	MUC2	2	14.46 (1.71-121.97)	Random	2.46	0.01	59	0.12
	MUC5AC	3	1.41 (0.85-2.34)	Fixed	1.32	0.19	0	0.62

Gender (male vs. female)	MUC1	7	1.10 (0.86-1.41)	Fixed	0.77	0.44	0	0.75
	MUC2	7	0.87 (0.68-1.12)	Fixed	1.07	0.29	8	0.29
	MUC5AC	6	0.93 (0.69-1.24)	Random	<0.00001	1.00	55	0.005

Tumor size (large vs. small)	MUC1	4	0.77 (0.53-1.12)	Fixed	1.38	0.17	19	0.30
	MUC2	2	0.70 (0.47-1.05)	Fixed	1.73	0.08	0	0.39
	MUC5AC	2	0.80 (0.48-1.32)	Fixed	0.87	0.38	0	0.41

Histological grade (3 vs. 1 and 2)	MUC1	12	1.39 (0.87-2.21)	Random	1.39	0.16	66	0.0007
	MUC2	7	0.75 (0.56-0.99)	Fixed	2.02	0.04	44	0.10
	MUC5AC	5	1.44 (0.70-2.97)	Random	0.99	0.32	79	0.0007

RR: risk ratio; Random: random effects model; Fixed: fixed.

**Table 3:** Results of meta-regression analysis exploring the source of heterogeneity with OS and DFS/RFS.

Mucins phenotype	Covariates	Univariate analysis (OS)				Univariate analysis (DFS)	
		Coefficient	SE	*P* value	Coefficient	SE	*P* value
MUC1	Antibody	0.055	0.087	0.538	-0.142	0.882	0.883
	Cut-off value	0.0297	0.032	0.369	0.155	0.295	0.635
	TNM stage	0.365	0.324	0.281	0.773	1.106	0.535
	Country	0.048	0.462	0.323	0.155	0.295	0.635
	Year	-0.001	0.014	0.964	-0.077	0.115	0.552

MUC2	Antibody	-0.204	0.215	0.367	0.550	0.252	0.094
	Cut-off value	-0.027	0.043	0.552	-0.030	0.221	0.898
	TNM stage	-0.309	0.124	0.054	-0.270	0.838	0.763
	Country	0.007	0.048	0.891	0.180	0.050	0.023
	Year	0.036	0.030	0.264	0.108	0.030	0.022

MUC5AC	Antibody	0.464	0.269	0.135	-0.139	0.434	0.769
	Cut-off value	0.187	0.158	0.282	-0.248	0.193	0.288
	TNM stage	0.923	0.211	0.055	-0.652	0.961	0.546
	Country	0.250	0.240	0.339	-0.379	0.293	0.287
	Year	0.135	0.073	0.859	0.102	0.069	0.236

## Supplementary Materials

Supplementary Materials Table S1: quality assessment of the included studies.
