# Supplementary material for: Prognostic and Clinicopathological Significance of MUC Family Members in Colorectal Cancer: A Systematic Review and Meta-Analysis
Source: Gastroenterol Res Pract. 2019 Dec 20;2019:2391670. doi: 10.1155/2019/2391670 (PMC6942850; doi:10.1155/2019/2391670)
Supplement: Supplementary Materials — Table S1: quality assessment of the included studies. [file 2391670.f1.doc]

**Table S1** Quality assessment of the included studies.

| First author | Year | Selection^1^ | | | | Comparability^2^ | Outcome^3^ | | |  |
| --- | --- | --- | --- | --- | --- | --- | --- | --- | --- | --- |
|  |  | Representativeness of exposed cohort ★ | Selection of non-exposed cohort ★ | Ascertainment of exposure ★ | No primary outcome was present at start of study ★ | Comparable on confounder ★★ | Outcome Assessment ★ | Adequate follow-up★ | Loss to follow-up★ | Total Score |
| Adams | 2009 | 1 | 1 | 1 | 0 | 2 | 1 | 1 | 0 | 7 |
| Al-Maghrabi | 2019 | 1 | 1 | 1 | 1 | 1 | 1 | 1 | 0 | 7 |
| Baldus | 2004 | 1 | 1 | 1 | 1 | 2 | 1 | 1 | 0 | 8 |
| Baldus | 2000 | 1 | 1 | 1 | 0 | 2 | 1 | 1 | 1 | 8 |
| Betge | 2016 | 1 | 1 | 1 | 1 | 1 | 1 | 1 | 0 | 7 |
| Dawson | 1987 | 1 | 1 | 1 | 0 | 2 | 1 | 1 | 1 | 8 |
| Diaz | 2018 | 1 | 1 | 1 | 1 | 2 | 0 | 1 | 0 | 7 |
| Duncan | 2007 | 1 | 1 | 1 | 0 | 2 | 1 | 1 | 1 | 8 |
| Elzagheid | 2013 | 1 | 1 | 1 | 0 | 2 | 1 | 1 | 0 | 7 |
| Hiraga | 1998 | 1 | 1 | 1 | 0 | 2 | 1 | 1 | 1 | 8 |
| Imai | 2013 | 1 | 1 | 1 | 0 | 2 | 1 | 1 | 1 | 8 |
| Ionescu | 2014 | 1 | 1 | 1 | 0 | 2 | 1 | 1 | 0 | 7 |
| Kang | 2011 | 1 | 1 | 1 | 1 | 2 | 1 | 1 | 0 | 8 |
| Kasprzak | 2018 | 1 | 1 | 1 | 1 | 2 | 1 | 1 | 0 | 8 |
| Khanh | 2013 | 1 | 1 | 1 | 0 | 2 | 1 | 1 | 1 | 8 |
| Kimura | 2000 | 1 | 1 | 1 | 0 | 2 | 1 | 1 | 0 | 7 |
| Kocer | 2006 | 1 | 1 | 1 | 0 | 1 | 1 | 1 | 1 | 7 |
| Kocer | 2002 | 1 | 1 | 1 | 0 | 2 | 1 | 1 | 0 | 7 |
| Lennerz | 2016 | 1 | 1 | 1 | 0 | 2 | 1 | 1 | 1 | 8 |
| Manne | 2000 | 1 | 1 | 1 | 1 | 2 | 1 | 1 | 1 | 9 |
| Matsuda | 2010 | 1 | 1 | 1 | 0 | 2 | 1 | 1 | 0 | 7 |
| Matsuyama | 2010 | 1 | 1 | 1 | 0 | 2 | 1 | 1 | 1 | 8 |
| Perez | 2008 | 1 | 1 | 1 | 0 | 2 | 1 | 1 | 0 | 7 |
| Shanmugam | 2010 | 1 | 1 | 1 | 1 | 2 | 1 | 1 | 1 | 9 |
| Sun | 2018 | 1 | 1 | 1 | 1 | 2 | 1 | 1 | 1 | 9 |
| Streppel | 2012 | 1 | 1 | 1 | 0 | 1 | 1 | 1 | 1 | 7 |
| Wang | 2017 | 1 | 1 | 1 | 0 | 2 | 1 | 1 | 0 | 7 |
| Wang | 2016 | 1 | 1 | 1 | 0 | 1 | 1 | 1 | 1 | 7 |
| Xiao | 2013 | 1 | 1 | 1 | 1 | 2 | 1 | 1 | 0 | 8 |
| You | 2006 | 1 | 1 | 1 | 0 | 1 | 1 | 1 | 1 | 7 |
| Yu | 2007 | 1 | 1 | 1 | 0 | 1 | 1 | 1 | 1 | 7 |
| Zhang | 2008 | 1 | 1 | 1 | 0 | 2 | 1 | 1 | 1 | 8 |
| Zwenger | 2014 | 1 | 1 | 1 | 0 | 2 | 1 | 1 | 1 | 8 |

^1^ “Selection” defined as representativeness of cases, selection of controls, exposure ascertainment, and no death when investigation began.

^2^ “Comparability” defined as comparable on confounders.

^3^ “Outcome” defined as outcome assessment, adequate follow-up, and loss to follow-up rate
